# Preclinical Theranostic Profiling of [^64^Cu]Cu-Acetate in Prostate Cancer

**DOI:** 10.3390/molecules30193957

**Published:** 2025-10-02

**Authors:** Sadaf Ghanaatgar Kasbi, Martin Savard, Céléna Dubuc, Yves Dory, Brigitte Guérin, Fernand Gobeil

**Affiliations:** 1 Department of Pharmacology and Physiology, Faculty of Medicine and Health Sciences, Université de Sherbrooke, Sherbrooke, QC J1H 5N4, Canada; sadaf.ghanaatgar-kasbi@usherbrooke.ca (S.G.K.); martin.savard@usherbrooke.ca (M.S.); celena.dubuc@usherbrooke.ca (C.D.); 2 Institute of Pharmacology, Faculty of Medicine and Health Sciences, Université de Sherbrooke, Sherbrooke, QC J1H 5N4, Canada; yves.dory@usherbrooke.ca; 3 Department of Chemistry, Faculty of Sciences, Université de Sherbrooke, Sherbrooke, QC J1H 5N4, Canada; 4 Department of Medical Imaging and Radiation Sciences, Faculty of Medicine and Health Sciences, Université de Sherbrooke, Sherbrooke, QC J1H 5N4, Canada; 5 Institut de Recherche sur le Cancer de l’Université de Sherbrooke (IRCUS), Université de Sherbrooke, Sherbrooke, QC J1H 5N4, Canada

**Keywords:** copper-64, theranostics, prostate cancer, radiopharmaceuticals, mice

## Abstract

Copper plays a critical role in cancer biology, with tumor cells exhibiting abnormal copper metabolism that drives proliferation and tumor growth. A limited number of preclinical and clinical studies have reported promising theranostic potential of copper-based radionuclides, such as ^64^Cu, for both diagnostic imaging and targeted radiotherapy in diverse cancers, including prostate cancer (PCa). In this work, we evaluated the cellular uptake and antitumor efficacy of [^64^Cu]Cu-acetate using both cellular and animal models of PCa. Uptake assays revealed that ~70% of the administered dose (10 kBq) was internalized by PC-3 cells within 24 h, predominantly localizing to the cytoplasm, with around 9% detected in the nucleus. These results were corroborated by comparable natural Cu-acetate uptake levels (at equimolar dose) in PC-3 cells, as quantified by ICP-MS. Clonogenic assays revealed a dose-dependent reduction in survival following treatment with [^64^Cu]Cu-acetate (3 and 6 MBq), whereas its non-radioactive counterpart [^Nat^Cu]Cu-acetate, even at excess concentrations (10 µM), had no significant effect. Ex vivo biodistribution studies showed selective tumor accumulation/retention alongside expected hepatic uptake. Clear tumor visualization was achieved using μPET imaging with [^64^Cu]Cu-acetate (10 MBq iv). A single higher dose (65 MBq iv) effectively reduced tumor growth in a subcutaneous PC-3 xenograft mouse model, without systemic toxicity, as evidenced by stable body weight. Together, these results further support the theranostic potential of [^64^Cu]Cu in PCa.

## 1. Introduction

Copper (Cu) plays a vital role in numerous cellular processes, including the regulation of growth and proliferation [1,2]. Notably, cancer cells often exhibit altered Cu metabolism compared to normal cells, partly due to dysregulated expression of the high-affinity Cu transporter 1 (CTR1, also known as SLC31A1). CTR1 expression has been reported to vary across different malignancies, including prostate cancer (PCa), glioblastoma, melanoma, lung, breast, and liver cancers [3,4,5,6]. While some studies suggest that CTR1 may contribute to elevated intracellular Cu in tumors [7,8,9,10], its specific role in PCa remains unclear [6,11]. Nevertheless, these observations indicate that Cu accumulation in tumors likely involves multiple mechanisms, providing a rational for metabolic imaging using positron emission tomography (PET).

Indeed, Cu-based radionuclides have demonstrated promising theranostic potential for both diagnostic imaging and targeted radiotherapy in cancers, including PCa [1,12,13,14,15,16,17]. Among the various Cu radioisotopes available for cancer imaging and therapy [18], the cyclotron-produced Cu-64 ([^64^Cu]Cu) stands out as one of the most versatile [13,14,19]. This interest is largely driven by its favorable decay properties. Its β^+^ emission (17.8%) enables high-resolution PET imaging for tumor visualization [20,21], while its β^−^ decay (38.4%) and electron capture (EC, 43.5%) support targeted radionuclide therapy or endoradiotherapy [18].

The therapeutic efficacy of [^64^Cu]Cu is closely linked to its nuclear localization and the emission of short-range, high linear energy transfer Auger electrons during EC decay, which can directly damage DNA [22,23]. In cancer cells, nuclear and perinuclear accumulation may be facilitated by endogenous Cu-binding proteins such as Atox1, a key player in intracellular Cu trafficking [24,25]. This targeting mechanism could enhance the radiobiological impact of [^64^Cu]Cu^2+^, particularly in tumors with elevated levels of Atox1 or related Cu-transport pathways.

Additionally, its relatively long half-life of 12.7 h, although shorter than that of commonly used therapeutic radionuclides such as ^177^Lu, ^90^Y, ^131^I or ^161^Tb, provides a suitable time window for both imaging and therapeutic evaluation in preclinical and early translational settings [20]. Its hepatobiliary excretion also helps reduce background signal in the pelvic region, resulting in improved imaging contrast [13,26]. While high activity levels required for radiotherapy may pose a risk of hepatic radiotoxicity [13], efficient clearance and the liver’s regenerative capacity are expected to mitigate this concern [27].

Together, these features contribute to a high tumor-to-background ratio, positioning [^64^Cu]Cu as a promising agent for both detection and treatment of PCa [28].

However, experimental evidence supporting the theranostic potential of [^64^Cu]Cu ions, and particularly their therapeutic efficacy in PCa, remains limited. This study aims to build on prior observations by providing new data on the uptake, biodistribution, and notably, the therapeutic impact of [^64^Cu]Cu^2+^ in PCa models.

## 2. Results

### 2.1. Cellular Accumulation and Subcellular Localization of [^64^Cu]Cu-Acetate in PC-3 Cells

**Figure 1:**
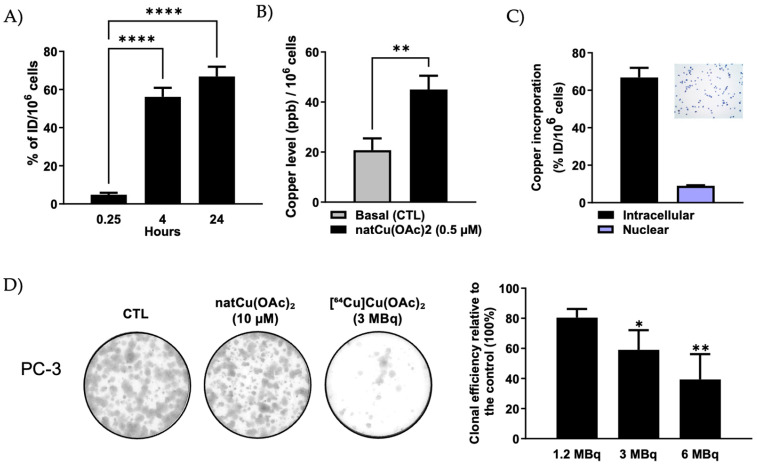
Cellular uptake, subcellular distribution, and radiotoxic effects of [^64^Cu]Cu-acetate, in PCa cells. (**A**) Time-dependent uptake of [^64^Cu]Cu-acetate (10 kBq) in PC-3 cells at 0.25 h, 4 h, and 24 h post-incubation. Data are expressed as a percentage of the injected dose per 10^6^ cells (%ID/10^6^ cells). A significant increase in uptake was observed at 4 h and 24 h compared to 0.25 h (**** *p* < 0.0001). *n* = 4. Statistical analysis was performed using ordinary one-way ANOVA with Dunnett’s multiple comparisons test. (**B**) Quantification of Cu accumulation in PC-3 cells following 24 h treatment with ^Nat^Cu-acetate (0.5 µM), measured by ICP-MS. A significant increase in intracellular Cu was observed compared to basal levels (** *p* < 0.01, unpaired *t*-test). *n* = 7–8. (**C**) Subcellular distribution of [^64^Cu]Cu-acetate in PC-3 cells after 24 h. Cu incorporation was predominantly cytoplasmic, with ~9% nuclear accumulation. Nuclear fraction purity was validated by optical microscopy following trypan blue staining (inset). *n* = 4. (**D**) Clonogenic survival of PC-3 cells following exposure to increasing activities of [^64^Cu]Cu-acetate (1.2, 3, and 6 MBq). Representative colony formation images (**left**) and quantification of clonal efficiency relative to untreated control (CTL; 100%) are shown (**right**). Data are presented as mean ± SEM from four independent experiments. Statistical analysis was performed using one-way ANOVA followed by Dunnett’s test. * *p* < 0.05, ** *p* < 0.01 vs. control.

To evaluate the kinetics of [^64^Cu]Cu-acetate ([^64^Cu]Cu(OAc)_2_) uptake in PC-3 cells, total cell-associated radioactivity was measured at multiple time points and expressed as the percentage of the injected dose per 10^6^ cells (%ID/10^6^ cells) (Figure 1A). A significant time-dependent increase in cellular accumulation was observed. Uptake was minimal at 15 min (4.8% ID/10^6^ cells) but increased significantly at 4 h (56.6% ID/10^6^ cells) and further at 24 h (66.8% ID/10^6^ cells) (**** *p* < 0.0001). These findings indicate a progressive and efficient uptake of [^64^Cu]Cu-acetate in PCa cells over time. Uptake of nonradioactive natural Cu-acetate (^Nat^Cu-acetate; [^63/65^Cu]Cu-acetate) was also evaluated using inductively coupled plasma–mass spectrometry (ICP-MS) following treatment of PC-3 cells with 0.5 µM for 24 h, to validate the previous findings (Figure 1B). Treated cells showed a significant increase in Cu content, up to ~45 ppb per 10^6^ cells compared to untreated controls, which showed baseline Cu levels of ~20 ppb per 10^6^ cells, most likely due to prior exposure to Cu present in the serum-containing culture medium (RPMI with 10% FBS: ~200 nM Cu; present study). This high intracellular level of Cu indicates the ability of PC-3 cells to maintain exogenous Cu. The consistency between γ-counter and ICP-MS results underscores the time-dependent uptake of [^64^Cu]Cu-acetate in PC-3 PCa cells. To further evaluate intracellular distribution, subcellular fractionation was performed at the 24 h time point. Trypan blue staining confirmed the purity of the nuclear fraction. Quantitative analysis revealed that most of the radioactivity was retained in the cytoplasmic compartment (~67% of total cell-associated ^64^Cu-acetate), with only a small fraction localized to the nucleus (~9%) (Figure 1C).

### 2.2. In Vitro Radiocytotoxicity of [^64^Cu]Cu-Acetate in PC-3 Cells

Clonogenic assays were conducted to assess the cytotoxic effects of [^64^Cu]Cu-acetate on PC-3 PCa cells (Figure 1D). Clonal efficiency was assessed following treatment with increasing doses of ^64^Cu-acetate (1.2, 3, and 6 MBq) and compared to untreated controls. A clear dose-dependent decline in clonal survival was observed. At 3 MBq, a significant decrease in colony formation was detected (*p* < 0.05), which was further pronounced at 6 MBq (*p* < 0.01). Notably, there was no detectable effect on clonogenic efficiency when PC-3 cells were treated with the stable, non-radioactive counterpart, ^Nat^Cu-acetate even at a higher concentration (10 µM).

### 2.3. Ex Vivo Biodistribution of [^64^Cu]Cu-Acetate and [^64^Cu]CuCl_2_ in PC-3 Tumor Xenograft-Bearing Nude Mice

A comparative biodistribution study was performed to evaluate the tissue distribution of ^64^Cu from its acetate and chloride salt forms in male nude mice bearing subcutaneous PC-3 tumors. One-hour post-injection (0.19–0.37 MBq; 5–10 µCi), the liver and kidneys exhibited the highest uptake among all organs, as shown in Table 1. Liver accumulation was highest for both ^64^Cu-acetate (31.56 ± 11.33% ID/g) and ^64^Cu-chloride (29.98 ± 4.69% ID/g), indicating hepatic clearance as the primary excretion route. Both radiotracers also showed notable kidney uptake (9.26 ± 2.52 and 9.66 ± 1.65% ID/g, respectively), suggesting a secondary but limited role for renal elimination. Although CTR1, the major Cu transporter, is expressed in the brain [29], brain uptake was the lowest among all organs tested, in line with previous findings [5,30]. This minimal accumulation is likely due to the inability of ^64^Cu to cross the intact blood–brain barrier. A trend toward higher tumor uptake was observed for ^64^Cu-chloride compared to ^64^Cu-acetate (5.02 ± 5.18 vs 1.96 ± 0.21% ID/g), but the difference was not statistically significant (*p* > 0.05).

### 2.4. In Vivo PET/CT Imaging of [^64^Cu]Cu-Acetate in PC-3 Xenograft-Bearing Nude Mice

**Figure 2:**
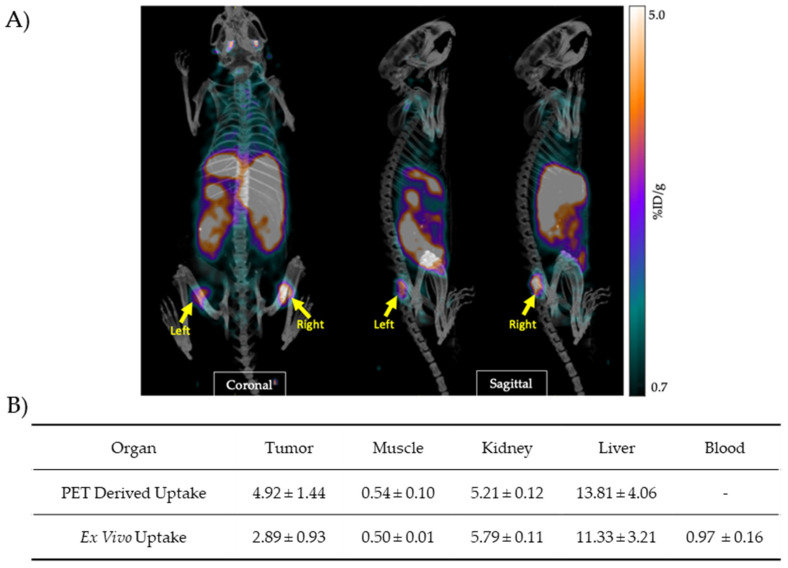
PET imaging and biodistribution of [^64^Cu]Cu-acetate in PC-3 tumor-bearing mice. (**A**) Representative coronal and sagittal PET/CT images acquired 24 h post-injection of [^64^Cu]Cu-acetate (7 MBq) in mice bearing subcutaneous PC-3 tumors. Yellow arrows indicate the locations of left and right tumors. (**B**) Quantitative uptake values (% ID/g) of [^64^Cu]Cu-acetate in selected tissues of PC-3 tumor–bearing mice, measured 24 h post-injection by in vivo PET imaging and ex vivo gamma counting performed on the same animals. Data are presented as mean ± SD (*n* = 3).

PET/CT images of [^64^Cu]Cu-acetate showed clear radiotracer accumulation in both the left and right PC-3 tumors (Figure 2A). Coronal and sagittal scans demonstrated symmetric uptake in subcutaneous flank tumors, with low background signal in surrounding tissues. Static PET scans were used to evaluate the in vivo distribution of ^64^Cu-acetate in PC-3 tumor-bearing nude mice. To further assess tissue distribution, ex vivo gamma counting was performed on the same animals following PET imaging. Organs were collected and analyzed for radioactivity content, enabling direct comparison between imaging-derived uptake and actual tissue radioactivity. As summarized in Figure 2, biodistribution analysis showed tracer accumulation in the liver and kidneys, consistent with the hepatic and renal clearance reported for [^64^Cu]CuCl_2_ [9]. The quantitative uptake values obtained ex vivo (% ID/g) corroborated the in vivo PET measurements, particularly in muscle and kidney, while slight variations were observed in tumor and liver values (Figure 2B). Minor differences in tumor and liver uptake likely reflect regional heterogeneity or differences in partial volume of interest. Interestingly, tumor uptake of [^64^Cu]Cu-acetate increased over time (1 h vs. 24 h), concomitant with a reduction in peripheral organ uptakes, suggesting enhanced tumor selectivity and prolonged retention in tumor tissue relative to non-target tissues (see Table 1 and Figure 2B).

### 2.5. Therapeutic Effect of [^64^Cu]Cu-Acetate on Tumor Growth in PC-3 Tumor-Bearing Mice

**Figure 3:**
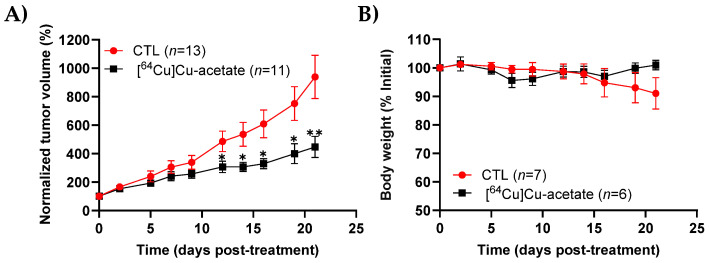
Therapeutic efficacy and tolerability of [^64^Cu]Cu-acetate in PC-3 tumor-bearing mice. (**A**) Tumor growth curves of mice treated with [^64^Cu]Cu-acetate or saline vehicle control (CTL). Treatment significantly delayed tumor progression compared with control. Data represent the mean ± SEM from 11–13 tumors per group (*n* = 6–7 mice/group); * *p* < 0.05, ** *p* < 0.01, unpaired Student’s *t* test with Welch’s correction. (**B**) Body weight monitoring over the study period showed no significant weight loss in either group (*n* = 6–7 mice/group). Data are presented as mean ± SEM. Control group data previously reported in [31], under identical conditions (*n* = 16 vs. *n* = 13).

The therapeutic effect of [^64^Cu]Cu-acetate was assessed in male nude mice bearing subcutaneous PC-3 xenografts following a single intravenous dose (65 MBq; 1.5–1.75 mCi) (Figure 3). Tumor growth was monitored and compared to that in a saline-treated control group. While tumors continued to grow in both groups, mice treated with ^64^Cu-acetate showed a significantly slower rate of tumor progression (Figure 3A). By day 22, tumor volume in the control group had increased nearly tenfold, whereas growth in the [^64^Cu]Cu-acetate treated group remained substantially limited, supporting a therapeutic effect. To evaluate potential systemic toxicity, body weight was measured throughout the study (Figure 3B). No significant weight loss was observed in the [^64^Cu]Cu-acetate group compared to controls, suggesting that the treatment was well tolerated.

## 3. Discussion

To date, few studies have explored the dual use of unchelated ^64^Cu as both a PET imaging agent and a therapeutic radionuclide, with most existing data based on ^64^Cu-chloride. Our study reinforces and broadens this body of evidence by showing that ^64^Cu-acetate can likewise fulfill both functions in PCa.

At the cellular level, [^64^Cu]Cu-acetate exhibited rapid and substantial internalization of ~67% ID/10^6^ cells at 24 h, with predominant cytoplasmic distribution. Cellular uptake at 24 h was comparable to levels observed at 4 h, indicating a prolonged intracellular retention time. Notable nuclear localization was observed at 24 h post-injection, with ~9% of the activity detected in the nuclear fraction. Similar findings were reported in PC-3 cells exposed to [^64^Cu]CuCl_2_, where cytoplasmic predominance and limited nuclear uptake were observed after 3 h of exposure [11]. This could reflect preferential trafficking of ^64^Cu toward vesicular and trans-Golgi pathways mediated by ATP7A and ATP7B [32]. Such compartmentalization may explain the cytoplasmic and nuclear uptake values of ^64^Cu observed in our study.

Functionally, [^64^Cu]Cu-acetate significantly reduced clonogenic survival in the androgen-independent, castration-resistant PC-3 cells in a dose-dependent manner. These findings are in agreement with previous work by Guerreiro et al., who reported that [^64^Cu]CuCl_2_ (2.8 MBq) inhibited clonogenic survival in PC-3 cells while sparing non-tumoral prostate cells (RWPE-1) [11].

Ex vivo organ biodistribution revealed similar tumor uptake and clearance profiles for [^64^Cu]CuCl_2_ and [^64^Cu]Cu-acetate, with both tracers showing dual hepatobiliary and renal elimination, and predominant liver clearance. The comparable tumor uptake observed with these two kit-ready Cu formulations likely reflects rapid in vivo binding of free Cu^2+^ to plasma proteins, such as albumin, ceruloplasmin, and transcuprein, resulting in similar biodistribution and dosimetry between the two formulations [33,34]. Thus, the primary advantage of using the acetate form would be pharmaceutical rather than dosimetric: acetate formulations are neutral and isotonic, whereas CuCl_2_ is typically obtained in 0.1–1 M HCl and requires buffering before administration (IAEA guidelines [35]). Accordingly, the acetate form offers formulation benefits without altering the radiation burden. Moreover, in accordance with the ex vivo findings, [^64^Cu]Cu-acetate enabled clear visualization of subcutaneous PCa xenografts using µPET imaging, with % ID/g values (2–5% ID/g at 24 h) comparable to those reported in previous studies (~4–6% ID/g at 24 h) [9,10].

In the context of endoradiotherapy, earlier preclinical studies using [^64^Cu]CuCl_2_ in glioblastoma (human U87-MG) and melanoma (mouse B16F10 or human A375M) xenografts models demonstrated marked tumor growth delay, survival benefit, and selective tumor uptake of [^64^Cu]Cu [4,5]. Our data confirms that the therapeutic potential of [^64^Cu]Cu extends beyond the tumor types and is not dependent on the specific formulation used. Notably, even a single administered dose of [^64^Cu]Cu-acetate effectively reduced PCa growth without inducing weight loss or acute toxicity. These results support the feasibility of Cu-based radiotherapy in PCa and open the door for multi-dose treatment protocols, which based on prior success in glioblastoma models [5], may further improve therapeutic outcomes. As noted in the Introduction, although concerns have been raised regarding radiation exposure to non-target organs—particularly the liver [9,10], a major site of Cu accumulation, clinical data to date suggest a favorable safety profile of [^64^Cu]Cu-based agents [13,14,30]. Furthermore, the efficient clearance of [^64^Cu]Cu, combined with the liver’s natural regenerative capacity, is anticipated to minimize the risk of hepatotoxicity.

A theranostic approach using [^64^Cu]Cu may be particularly valuable in PCa cases that lack established molecular targets such as PSMA or GRPR, as well as in those involving emerging receptor biomarkers currently under investigation (e.g., NTR1, NPR-A, B1R) [31,36,37]. As noted by Cantiello et al. [38], up to 10% of PCa cases, including aggressive subtypes like neuroendocrine PCa, do not express PSMA or standard epithelial markers, leading to false-negative results on PSMA-targeted PET imaging and complicating diagnosis and staging. A recent triple-tracer PET imaging study in patients with metastatic PCa revealed that ~53% would have qualified for PSMA radioligand therapy, while none were eligible for DOTATATE-based radiotherapy [39]. Similarly, GRPR-targeted agents such as NeoBOMB1 face limitations due to heterogeneous GRPR expression in PCa. In this context, [^64^Cu]Cu PET/CT offers a promising alternative as a complementary imaging and therapeutic tool in cases refractory to standard molecular imaging approaches. Its tumor uptake is driven by Cu metabolism and transporter expression, which may remain active even in PSMA- or GRPR-negative lesions. While intratumoral heterogeneity also affects Cu-based tracers, preclinical studies have shown that ^64^Cu uptake can vary across and within tumor models, highlighting the need for individualized assessment [40,41].

In conclusion, pharmaceutical grade [^64^Cu]Cu exhibits potential as a *bona fide* theranostic agent, with encouraging results for both PET imaging and targeted radiotherapy of PCa. Nonetheless, further studies are required to validate its clinical utility and to optimize its application in therapeutic settings.

## 4. Materials and Methods

### 4.1. Production of [^64^Cu]Cu

[^64^Cu]Cu was produced via the ^64^Ni(p,n)^64^Cu nuclear reaction using a TR-19 or TR-24 cyclotron (ACSI, Advanced Cyclotron Systems, Inc., Richmond, BC, Canada) and an electroplated ^64^Ni-enriched target on a rhodium disc. Following the previously described procedure [42], the resulting [^64^Cu]CuCl_2_ was recovered in 0.1 M HCl. [^64^Cu]Cu-acetate was then prepared by dissolving [^64^Cu]CuCl_2_ in 0.1 M ammonium acetate buffer (pH 5.5). The apparent molar activity (AMA) of the resulting [^64^Cu]Cu-acetate, averaging 350 ± 40 MBq/nmol, was determined by ligand titration following the method of McCarthy et al. [43], using NOTA (1,4,7-triazacyclononane-N,N′,N″-triacetic acid) as the chelator in place of TETA (1,4,8,11-tetraazacyclotetradecane-N,N′,N″,N‴-tetraacetic acid). Briefly, serial dilution titrations were performed by adding increasing amounts of NOTA to aliquots containing a fixed amount of [^64^Cu]Cu under standardized radiolabeling conditions (pH ~5.5, room temperature, 15 min). Complexation efficiency was assessed by radio-TLC, and the lowest concentration of NOTA yielding ≥95–97% complexation was used to calculate the AMA. This calculation assumes a 1:1 chelation stoichiometry and provides an estimate of the amount of non-radioactive (carrier) copper present in the sample. The resulting [^64^Cu]Cu was reconstituted in phosphate-buffered saline (PBS, pH 7.4) for in vitro studies or in isotonic saline for in vivo studies.

### 4.2. Cell Cultures

The human prostate adenocarcinoma cell line PC-3 was obtained from the American Type Culture Collection (ATCC, Manassas, VA, USA). The cells were cultured in RPMI-1640 medium (Wisent Inc., Saint-Jean-Baptiste, QC, Canada) supplemented with 10% heat-inactivated fetal bovine serum (FBS, Wisent Inc.) and 1% penicillin/streptomycin (Pen/Strep). Cultures were maintained at 37 °C in a humidified 5% CO_2_ incubator. For passaging, adherent cells were detached using 0.05% trypsin/EDTA (Wisent Inc.) or StemPros Accutase^®^ (Life Technologies, Carlsbad, CA, USA), with selection based on experimental requirements.

### 4.3. In Vitro Cellular Uptake and Nuclear Localization of [^64^Cu]Cu-Acetate

Cellular uptake and nuclear localization studies were performed by incubating PC-3 cells with [^64^Cu]Cu-acetate (10 kBq) for 0.25 h, 4 h, or 24 h at 37 °C. At each time point, the culture medium was collected, and the cells were washed with Hank’s balanced salt solution (HBSS, Wisent Inc). Whole cells were collected using Accutase, and nuclei were isolated according to the previously described methods [37,44]. Radioactivity in the medium, whole cells, and nuclei was quantified using a gamma counter (Cobra II auto-gamma counter, Packard, Minneapolis, MN, USA). Nuclear uptake was calculated as the percentage of radioactivity in purified nuclei relative to the total cellular radioactivity.

### 4.4. Cell Uptake Analysis of ^Nat^Cu-Acetate Measured by ICP-MS

PC-3 cells were incubated for 24 h at 37 °C with 500 nM ^Nat^Cu-acetate (Sigma-Aldrich; 229601, Burlington, MA, USA) or left untreated as a control in RPMI-1640 medium with 1% FBS. Following washing and cell harvesting, pellets were digested in a HNO_3_/H_2_O_2_ 50:50 mixture and analyzed for Cu content using inductively coupled plasma-mass spectrometry (ICP-MS, PerkinElmer Elan DRC II, Woodbridge, ON, Canada). Samples and calibration standards were diluted with 2% HNO_3_/0.4% H_2_O_2_ prior to analysis.

### 4.5. Clonogenic Assay

PC-3 cells were seeded in a 6-well plate and treated 48 h later with different doses (1.2, 3, and 6 MBq; corresponding to about 2.5, 5 and 10 nM, respectively) of [^64^Cu]Cu-acetate or with 5 µM of ^Nat^Cu-acetate. The cells were then incubated at 37 °C for 10–14 days to allow colony formation. After incubation, colonies were stained and counted [45].

### 4.6. Subcutaneous Tumor Xenograft Model

PC-3 xenograft tumors were established in male Nu/Nu nude mice (Crl:NU-Foxn1nu: 4–6 weeks old; Charles River Laboratories, Inc., Wilmington, MA, USA) by subcutaneous injection of 2.5 × 10^6^ cells suspended in 50 μL of a 1:1 mixture of serum-free medium and Matrigel (#356234, Sigma-Aldrich, Burlington, MA, USA) into both flanks. Within 10–12 days post-inoculation, the tumors reached the appropriate size, after which the mice were used for ex vivo and in vivo studies.

### 4.7. Biodistribution and PET Imaging in PC-3 Xenograft Mice

Biodistribution of [^64^Cu]Cu-acetate and [^64^Cu]Cu-chloride was performed in PC-3 tumor-bearing nude mice through intravenous injection of 5–10 µCi in the caudal vein under isoflurane anesthesia. At 1 h post-injection, isoflurane-anesthetized mice were euthanized by 5% CO_2_ inhalation. Organs were collected, washed in 0.9% saline, and radioactivity was measured using a Hidex automated gamma counter (Cobra II auto-gamma counter, Packard, Minneapolis, MN USA). Tissue radioactivity was quantified and expressed as percentage injected dose per gram (% ID/g), using external ^64^Cu standards to account for physical decay. In vivo distribution of [^64^Cu]Cu-acetate was further evaluated in PC-3 xenograft-bearing nude mice by small-animal PET imaging using a LabPET8 scanner (Gamma Medica–IDEAS Inc.; 7.5-cm axial and 10-cm transaxial field of view, Northridge, CA, USA), in conjunction with X-ray CT acquired on a MILabs U-CT system (Utrecht, The Netherlands) for anatomical correlation [46]. For PET imaging, 6–9 MBq in 200 μL of [^64^Cu]Cu-acetate was administered via the tail vein of the animal, and a 30-min static acquisition was performed 24 h post-injection, followed by a CT scan. The percentage of injected dose per gram (% ID/g) of tissues was calculated using a calibration phantom loaded with a known activity concentration [47]. Tumor uptake was quantified by placing a volume of interest (VOI) over the central region of the tumor, and the mean activity was calculated from voxels within the upper quartile of signal intensity. To validate PET findings, the same animals were euthanized following imaging, and ex vivo biodistribution was performed as described.

### 4.8. Radiotherapy with [^64^Cu]Cu-Acetate in PC-3 Xenograft Mice

The therapeutic efficacy of [^64^Cu]Cu-acetate was investigated in PC-3 xenograft-bearing mice. When the tumor reached a diameter of 50–200 mm^3^, the mice were randomly divided into two groups (6–7 animals per group). One group received 65 MBq (1.5–1.75 mCi) of [^64^Cu]Cu-acetate, while the other group was considered the control and received an equivalent volume of isotonic saline (100 µL). Although no dosimetry was performed, the radiation dose used was based on previous studies and falls within the typical range for ^64^Cu-based internal radiotherapy in mice [4,48,49]. Tumor volume was measured with a digital vernier caliper and calculated using the formula: length × width^2^. Normalized tumor growth was calculated by dividing tumor volumes at each time point by the initial volume measured on the day of treatment. The body weight of the mice was measured every 2 days to assess any potential toxicity. A tumor diameter of 1000 mm^3^, the development of an ulcerated necrotic area within the tumor, and any movement restrictions or weight loss exceeding 20% were considered endpoints for study termination.

### 4.9. Statistical Analyses

Statistical analyses were performed using GraphPad Prism software (version 10.2; GraphPad Software, San Diego, CA, USA). Data are reported as mean ± SEM or mean ± SD, as specified. Group comparisons were performed using one-way ANOVA followed by Dunnett’s test, or an unpaired Student’s *t* test, as appropriate. A *p* value < 0.05 was considered statistically significant.

## Figures and Tables

**Table 1:** Ex vivo biodistribution of [^64^Cu]Cu(OAc)_2_ and [^64^Cu]CuCl_2_ in PC-3 xenograft mice at 1 h post-injection (p.i.).

Organ	Blood	Plasma	Kidney	Pancreas	Liver	Heart	Lung	Tumor	Muscle	Bone	Brain
[^64^Cu]Cu(OAc)_2_	1.95 ± 0.65	2.96 ± 0.86	9.26 ± 2.52	3.15 ± 1.18	31.56 ± 11.33	2.84 ± 0.43	4.19 ± 1.49	1.96 ± 0.21	0.61 ± 0.13	0.81 ± 0.08	0.36 ± 0.10
[^64^Cu]CuCl_2_	3.23 ± 1.97	2.28 ± 0.44	9.66 ± 1.65	2.35 ± 0.41	29.98 ± 4.69	3.52 ± 1.35	5.64 ± 3.78	5.02 ± 5.18	0.64 ± 0.16	1.11 ± 0.59	0.34 ± 0.08

Values are expressed as mean ± SD (*n* = 3).

## Data Availability

All data generated or analysed during this study are available from the corresponding authors upon reasonable request.

## Abbreviations

CTR1	Copper transporter 1
FBS	Fetal bovine serum
PBS	Phosphate-buffered saline
HBSS	Hank’s balanced salt solution
ICP-MS	Inductively coupled plasma–mass spectrometry
PCa	Prostate cancer
Pen/Strep	Penicillin/Streptomycin
PET	Positron emission tomography
